# Obstructive lung diseases and inhaler treatment: results from a national public pragmatic survey

**DOI:** 10.1186/1465-9921-14-94

**Published:** 2013-09-22

**Authors:** Fulvio Braido, Ilaria Baiardini, Massimo Sumberesi, Francesco Blasi, Giorgio Walter Canonica

**Affiliations:** 1 Allergy and Respiratory Diseases Clinic, DIMI, University of Genoa, IRCCS AOU San Martino-IST, Genoa, Italy; 2 Managing Director, Doxa Marketing Advice, Milan, Italy; 3 Department of Pathophysiology and Transplantation, Università degli Studi di Milano, IRCCS Fondazione Ca’ Granda, Ospedale Maggiore Policlinico, Via Francesco Sforza, Milan 35, 20122, Italy

## Abstract

**Background:**

The opinions held by the general population on obstructive lung disease and inhaler devices could influence asthma and chronic obstructive pulmonary disorder (COPD) management and treatment adherence.

The aim of the present public pragmatic survey was to evaluate the opinions, beliefs and perceptions of Italian people with respect to respiratory diseases as well as their perspectives on the use of inhaler devices.

**Methods:**

This survey was conducted on a group of 2,008 individuals forming a representative sample of the Italian population aged 15 years and over. It was based on personal interviews that were administered in the homes of the respondents using a structured questionnaire that took approximately 30 minutes.

**Results:**

Awareness of obstructive lung diseases is poor. Asthma, but not COPD, was perceived as a common and increasingly prevalent disease by the majority of the interviewees. Allergy, pollution and smoking were considered to be responsible for both of these diseases. The rates at which the respondents claimed to be suffering from asthma and COPD were lower than expected (4% and 2%, respectively). Inhaled drugs were recognised as the main treatment by 65% of the respondents. The great majority of respondents attributed positive characteristics to the inhaler device (e.g., safety, reliability, effectiveness, ease of use and practicality). Compared to people who have never used inhaler devices, individuals who suffer from asthma or COPD were more confident in their use and showed a greater belief in their safety, reliability and trustworthiness. People older than 64 years showed less attention to the properties of these devices.

**Conclusions:**

The present results highlight the need for public interventions aimed at improving awareness of obstructive lung disease and reveal various potentialities and critical issues for inhaler device usage. Switching of devices was considered feasible by most of the interviewees, as long as the choice is carefully explained by their physician.

## Background

Chronic respiratory diseases are among the leading causes of morbidity and mortality, and the prevalence of these diseases is expected to increase in the coming years [1-4]. Data published by the WHO suggest that hundreds of millions of people currently suffer from these conditions worldwide [4]. It is estimated that there are approximately 7 million asthma and COPD patients in Italy, comprising 11.5% of the general population. The prevalence of asthma is 6.2%, or a total of approximately 3.7 million patients. COPD has a prevalence of 5.3% (for a total of approximately 3.3 million cases) [5,6]. Every year, nearly 300,000 new cases of asthma and COPD are reported (an estimated incidence of 6%), and there are approximately 40.000 deaths yearly (6.8%) (22,329 men and 17,620 women) [7]. Italy spends 14 billion euros each year on respiratory diseases (equal to 1% of the Italian GDP); of these costs, 5 billion euros are for direct/indirect costs associated with asthma and 9 billion euros are for direct/indirect costs associated with emphysema and/or COPD. The weighted per capita cost of asthma therapy is equal to 1,434 euros, whereas it is 2,723 euros for COPD [8,9].

Although effective therapies exist for these conditions [10,11], chronic respiratory diseases are frequently not adequately treated. One of the main problems is a lack of adherence to treatment by patients [12-17].

The term ‘compliance’ (the extent to which a person’s corresponds to physician’s prescription) has been used for a long time to define the observation of the prescriptions (i.e. drugs, medical devices, dietary or behavioural prescriptions) by the patient. More recently, the term ‘adherence’ has been preferred over the concept of compliance because of the authoritative and paternalistic connotations of the latter one. Adherence has been defined as the extent to which a person’s behaviour corresponds to the recommendations agreed with a health care provider’. Adherence involves consumer’s choice and is intended to be non-judgemental, at variance with compliance, which reinforces patient passivity and blame [15].

The treatment of these diseases involves the administration of drugs using an inhaler device and specific techniques that the doctor must explain to the patient. During treatment, the patient may encounter a difficulty in using the device, leading to under-use and/or misuse [18,19]. This improper use has inevitable consequences on both the health of the patient and the entire health care system [20-22].

Relatively little research has addressed the public awareness of asthma, COPD and related treatment issues [23-28].

The aim of the present public pragmatic survey is to evaluate the following:

–opinions, beliefs and perceptions of Italian people regarding respiratory diseases.

–attitudes, motivations and barriers regarding the use of inhaler devices.

## Methods

This *ad hoc* survey was conducted on a sample of 2,008 individuals that represented the Italian population aged 15 years and over (52.1 million people).

When choosing the sample size, the need to accommodate a maximum margin of sampling error was taken into account. Considering the sample of 2,008 respondents out of an overall population of more than 52 million people aged >15 years, a sampling error of ± 2.88% is reported, which ensures a confidence level of 99% in the worst-case scenario of parameter estimation (p = 50%).

The survey was based on personal interviews that were administered in the homes of the respondents using a structured questionnaire that took approximately 30 minutes to complete and the support of a laptop computer (CAPI system, Computer-Assisted Personal Interviewing).

A total of 130 fully trained interviewers from the national network of DOXA interviewers who specialise in face-to-face interviews conducted the survey between May 22, 2012 and June 3, 2012.

The community under study was divided into sections or "layers" based on two criteria: the region and size of the municipality of residence.

The number of interviews conducted in each "layer" (i.e., in municipalities of Italian regions with populations of less than 5,000 residents) was calculated to ensure that the number of interviews was proportional to the distribution among the various "layers" of communities under study.

All of the units included in the sample represent a miniature reproduction of the community being considered (proportional stratified sampling method) according to the two above-mentioned criteria. The next step was to identify the sampling units within each "layer" (municipalities, municipality area and people). Among the municipalities, certain sampling points to perform the interviews were selected in each layer. An adequate number of polling stations (each of which corresponds to a specific area of the town) were selected in each municipality to ensure that all of the various types of residential areas within the town (the central areas, peripheral areas and isolated houses) were represented properly.

The names and addresses of the people to be interviewed were extracted from the electoral roll. People aged 15 to 17 years, who cannot yet register to vote, were selected using the quotas method.

During the processing phase, the data were weighted and a weight (or weighting factor) was assigned to each interview to perfectly balance the sample compared to the reference population. The weighting procedure was performed while considering the following variables: gender according to age, region according to town amplitude, education (degree/diploma/middle school/elementary school) and employment status (working/not working).

### Statistical analysis

A descriptive analysis of the answers to the questionnaires was performed, and the *χ*2 test was used to test for an association between the questionnaire answers and membership in a particular group (inhaler users/non-users or inhaler users younger/older than 64 years). The relationship between single items and the general opinion regarding inhaler devices (latent importance) was evaluated using the Pearson correlation coefficient. The level of accordance was calculated as the percentage of patients who agreed with each item.

## Results

In total, 2,008 individuals (52% females) were evaluated. The demographic characteristics of the population are given in Table 1.

**Table 1:** Sample demographics

**Demographic characteristics**		**%**
**Sex**	Males	48
	Females	52
**Age**	< 25 yrs.	12
	25-34 yrs.	15
	35-44 yrs.	19
	45-54 yrs.	17
	55-64	15
	> 64 yrs.	22
**Geographic area**	Northwest	27
	Northeast	19
	Centre	20
	South and Islands	34
**Education**	Primary and secondary school	50
	High school	41
	Academic degree	9
**Professional condition**	Employed	47
	Unemployed	53
**Socio**-**economic condition**	Low	2
	Medium-low	13
	Medium	70
	Medium-high	13
	High	2

To assess their awareness of the diseases under study, the respondents were asked whether they had recently heard of any of several diseases, including asthma, migraines, hypertension, diabetes, osteoporosis, arthrosis, colitis, hepatitis, celiac disease, glaucoma and COPD. Only 51% and 14% of the interviewees recognised “asthma” and “COPD”, which ranked fifth and last (eleventh), respectively.

**Figure 1:**
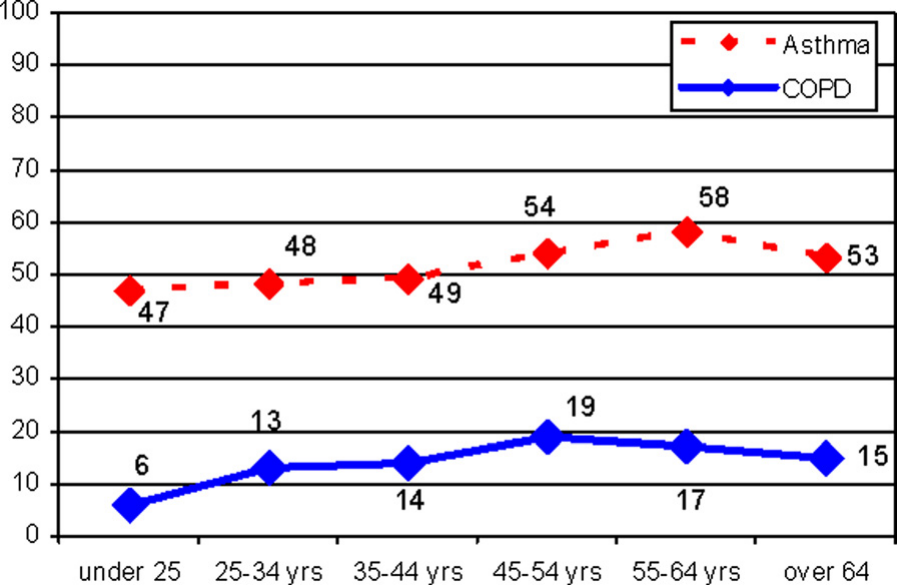
Awareness by age.

Considering the level of disease awareness according to the age, both of the pathologies were better known by individuals who were >45 years old. In particular, the level of asthma awareness was highest in the 55–64 year age group (58%), whereas COPD awareness was highest in the 45–54 year age group (19%). For both diseases, the level of awareness reported by people under 25 years of age was the lowest among the age groups (47% for asthma and 6% for COPD) (Figure 1).

Although 42% of the interviewees reported a medium-high level of asthma awareness, 14% declared that they knew nothing about the disease, whereas 33% and 40% of the respondents reported a poor and a fair knowledge of it, respectively. The survey revealed a severe lack of knowledge of COPD; only 13% of the interviewees reported a medium-high level of COPD awareness, whereas 47% reported that they knew nothing about the disease.

With respect to the perception of disease prevalence, 68% of the respondents indicated that asthma is highly widespread among the population, whereas only 21% indicated the same regarding COPD. Moreover, 50% and 16% of the sample considered asthma and COPD, respectively, to be increasing among the population, whereas 22% and 17% answered that the prevalence is stable.

Among the 1038 individuals who had previously stated they had heard of asthma, children, elderly people and people living in the city were considered the groups most likely to be at risk for asthma by 29%, 28% and 28% of the respondents, respectively. Among the 291 respondents who had previously stated they had heard of COPD, 27% of them considered the elderly to be most likely to suffer from COPD caused smoking, followed by people who worked in particular jobs (24%).

Allergies, pollution and smoking were considered to be the most relevant causes of asthma by 74%, 49% and 42% of the respondents, respectively. The same causes were also considered as the most likely to lead to the development of COPD by 35%, 35% and 49% of the respondents, respectively.

According to the survey, awareness of the disease burden in terms of symptoms was very high (92%) for both asthma and COPD, as was awareness of the serious impact of the disease on the lives of patients (82% for asthma and 87% for COPD).

The majority of the respondents (66% for asthma and 50% for COPD) believed that the most frequently used treatments for both diseases are inhaled drugs.

In the survey, 13% of the respondents indicated that they either personally suffer or have relatives suffering from asthma/COPD: 4% suffered from asthma and 2% suffered from COPD, whereas 6% and 1% had at least one relative suffering from asthma and COPD, respectively.

The survey showed that 65% of asthma/COPD sufferers used an inhaler device (25% regularly, 32% during the acute phases and 7% only for emergencies), whereas 16% had never used one and 19% had only used one in the past. Regarding familiarity with the inhaler device, 6% of the sample reported having had a direct experience with asthma/COPD. However, 24% of the respondents reported an indirect experience with the diseases: 7% had at least one relative suffering from asthma/COPD (78% of whom used a device), whereas 17% of the interviewees knew at least one person suffering from asthma/COPD (70% of whom used a device). Finally, 70% of the sample only had a nominal awareness of the diseases, as they did not know anyone suffering from asthma/COPD; of these respondents, 68% had heard about inhaler devices.

When the patients who use inhaler devices were asked whether their physician (GP and/or specialist) had explained to them how the device should be used, 89% of the respondents answered that they had been given an adequate and exhaustive explanation, 7% indicated that the explanation had been too generic and 4% indicated that no explanation had been provided to them.

The entire sample was questioned regarding attitudes towards inhaler devices in terms of efficacy, practicality and reliability/safety. The devices were considered to have a more rapid effect by 76% of the respondents, to act in a more precise way by 74%, to be more effective by 68% and to have a more prolonged effect over time by 50%, relative to other routes of drug administration. Furthermore, 79% of the respondents indicated that taking a drug through an inhaler device seems to be more practical and convenient; the same percentage felt completely capable of making an inhaler device work, and 73% of the respondents stated that using the inhaler device made it easier to follow a treatment plan. Moreover, 57% of the sample considered these devices not difficult to use in a daily routine. Regarding reliability/safety, 80% of the interviewees considered the devices to be absolutely safe and reliable, whereas 50% believed that taking a drug through an inhaler device can reduce the risk of side effects.

Among the respondents, 75% indicated that taking a drug through an inhaler device is the best solution for respiratory diseases, 58% indicated that it is also an excellent solution for non-respiratory disease and 45% only considered it to be a possible option for less serious diseases. Conversely, 33% of the respondents replied that using these devices carried the impression of following a mild treatment.

Considering the use of inhaler devices in relation to mood, the majority of the sample (74%) indicated that they felt completely calm and relaxed when thinking about using one of these devices, 50% did not believe that using it would contribute to the efficacy of the drug itself, 30% did not feel comfortable with the idea of using it and 61% believed that all of these devices are equal.

More than a third of the sample expressed concerns regarding the hygiene of the device. Conversely, a lower percentage of respondents expressed concerns regarding the possibility that the device could contain substances that are potentially harmful to the environment or harmful to their health, or that the devices could be made of harmful materials (23%, 22% and 19%, respectively). Other concerns included problems with the functioning of the device itself: 33% of the respondents indicated that when using the device, it is not clear when the drug has been taken, whereas 30% and 28% believed that the device may not work properly or that they may use it incorrectly without realising it. However, 79% of the respondents felt confident of their ability to use the device properly.

Additionally, 72% of the interviewees indicated that using an inhaler device ensured that they always received the same dose of the drug; however, 33% of the respondents indicated that using the device introduced the possibility that some of the drug could be wasted and not reach the lungs, and 25% of the respondents indicated that the dosage could be either insufficient or excessive.

The results indicated that 72% of the sample considered the inhaler device to be suitable for them; 71% stated that if they had to follow a treatment plan, they would rather take an inhaled drug rather than pills; 61% would be willing to use an inhaler device for a long period of time; and 56% would only be willing to use such a device for short period of time. Regarding their willingness to change inhaler devices, 86% of the sample wished to be informed of exactly how the new device works, 85% and 60% would change their device if their physician advised them to do so and 81% preferred to maintain their current type of inhaler device if they changed the drug they were taking.

The overall opinion of the respondents on inhaler devices was positive, especially among those who used the devices (94%), those who had a relative who used one (94%) and those who had at least heard about them (90%). The percentage of respondents with a positive opinion was lower (63%) among those who were not familiar with the devices.

**Figure 2:**
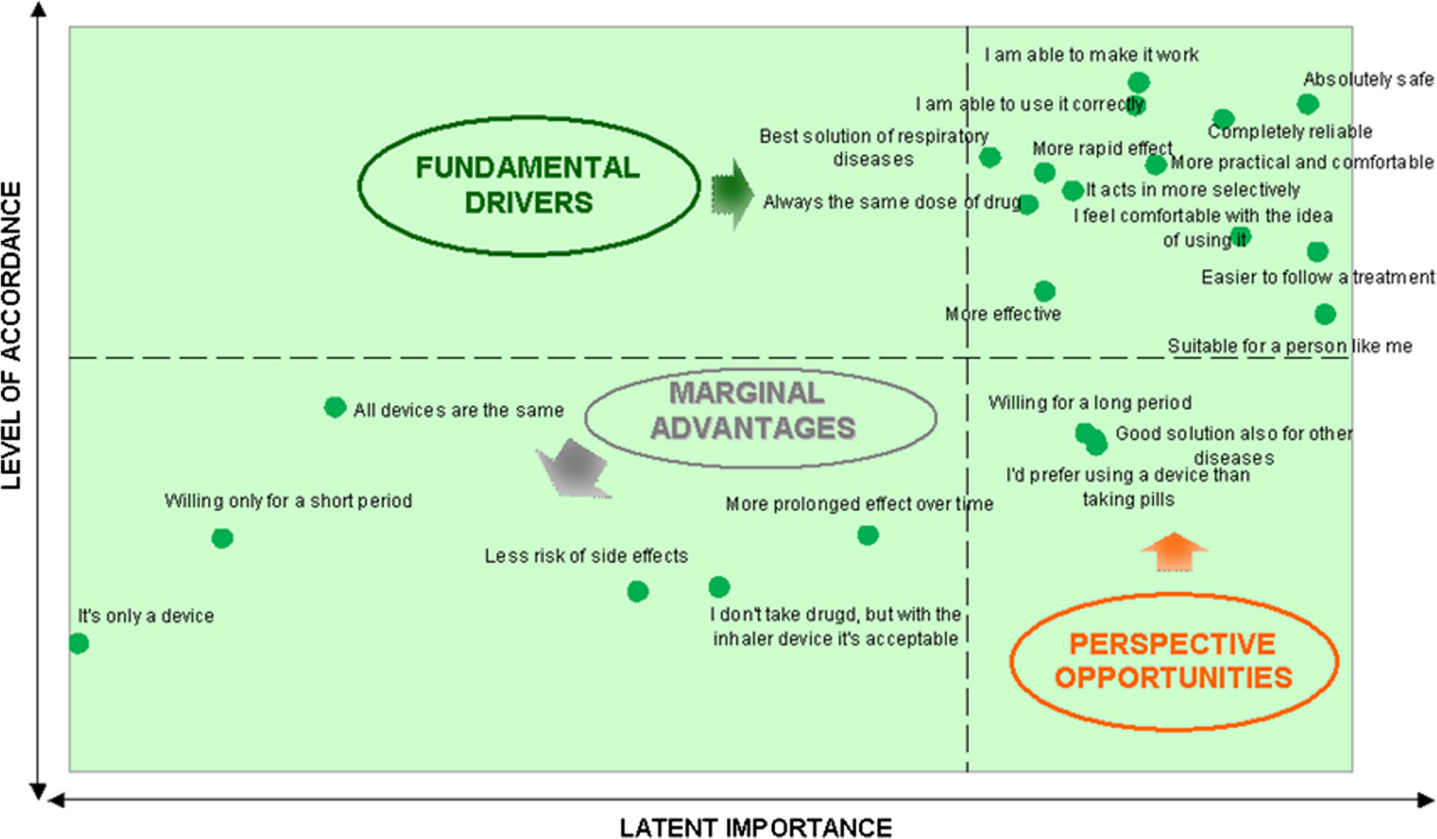
**Reasons for adherence: relationship between level of acceptance and latent importance.** The upper right side quadrant represents the reasons for adherence already achieved that need to be maintained; the perspective opportunities represent the reasons for adherence for which a return of investment is highly probable. The reasons for adherence, located in the left lower quadrant, represent the issues for which a return of investment is not as probable.

**Figure 3:**
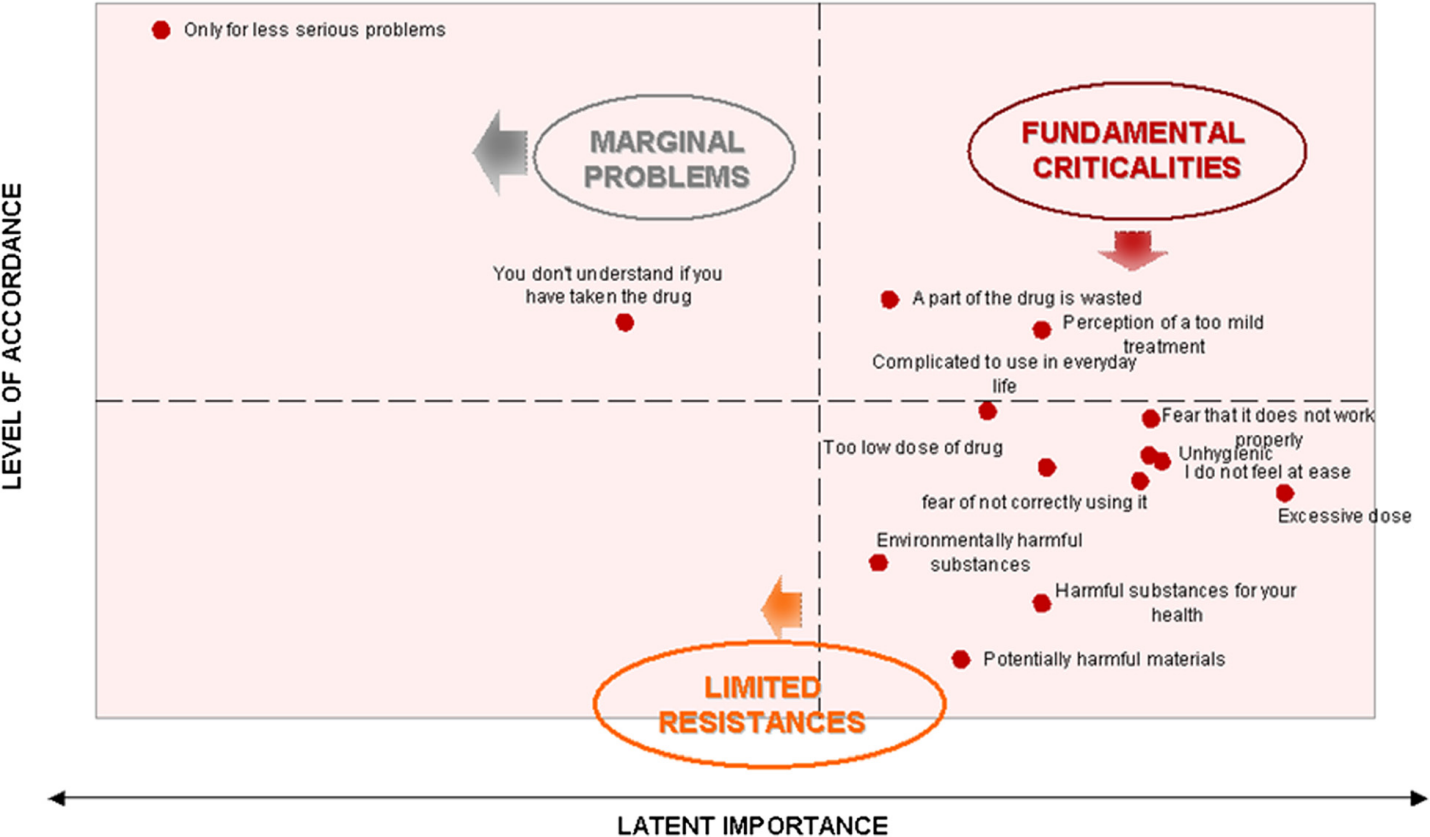
**Barriers for adherence: relationship between level of acceptance and latent importance.** The lower right side quadrant represents the barriers for adherence already contrasted that need to be maintained; the fundamental criticalities represent the barriers for adherence for which a return of investment is highly probable. The barriers for adherence, located in the left upper quadrant, represent the issues for with a return of investment is not as probable.

Strategic areas of intervention needed to improve adherence to inhaler device treatments are reported in Figure 2 and Figure 3.

Table 2 reports the significant differences revealed by comparing sufferers (6%) to non- sufferers (94%) and interviewees aged ≤64 years (76%) to those >64 years (24%).

**Table 2:** **Significant differences in answers between sufferers** (**S**) **and non**-**sufferers** (**NS**) **and interviewees aged** ≤**64 and** >**64 years**

		**I agree**			
		**S**	**NS**	***χ***^**2**^	**p**
**Motivation**	I would be willing to use the inhaler device for a long period for therapies that last several months	75.5	60	11.8613	p < 0.0027
	Using the inhaler device makes it easier to follow a treatment	84	72.1	10.8456	p < 0.0044
**Fear**	Using the inhaler device ensures that you always take the same dose of the drug	80.8	71.8	13.0236	p < 0.0015
	I fear that the dispenser may contain environmentally harmful substances	34.8	21.8	11.5824	p < 0.0031
	I think that a drug taken through an inhaler device is more effective	77.1	67.4	10.8163	p < 0.0045
**Sense of security**	I think that a drug taken through an inhaler device has a more prolonged effect over time	60.2	49.1	9.6056	p < 0.0082
		≤**64**	>**64**	***χ***^**2**^	**p**
**Trust**	I feel totally able to correctly use an inhaler device	80.6	70.6	21.6091	<0.0001
	I feel totally able to make an inhaler device work	81.2	72.4	20.0434	<0.0001
	The inhaler device seems to be an absolutely safe device	81.2	73.8	12.3264	<0.0021
**Availability**	I would only be willing to use the inhaler device for a short period of time	58.2	53.2	12.2936	<0.0021
**Fear**	Using the inhaler device introduces a risk of taking a very low dose of the drug	24.3	28.5	10.2285	<0.0060

***χ***^**2**^ Chi-square.

## Discussion

The data collected through this Italian public pragmatic survey revealed that the awareness of obstructive lung diseases is poor and that minimal differences exist among age classes. The opinions, beliefs and perspectives on asthma conflict with those on COPD. Asthma was perceived to be a common and increasingly prevalent disease by the majority of people interviewed, whereas the prevalence of COPD and its increase were recognised by only approximately 20% of the respondents. The difficulty in identifying individuals who are at risk for asthma and COPD was recognised by a high percentage of those interviewed. Three common factors, with some differences, were indicated as responsible for both asthma and COPD: allergies, pollution and smoking. Awareness of these factors may depend on media campaigns that have frequently dealt with these themes. The role of smoking as a cause of disease was recognised for both asthma and COPD by approximately half of the respondents. Allergies were considered to be the most relevant cause of asthma by three out of four respondents, and allergies were also indicated as a factor inducing COPD by 35% of the respondents. Pollution seemed to be more relevant for asthma than for COPD. The percentage of patients who claimed to suffer from asthma and COPD was lower than expected based on the prevalence of these conditions in Italy, as it has been estimated that over 3 million families are affected by these diseases [5,6]. Inhaled drugs were recognised as the most common treatment for asthma and COPD by 65% of the respondents. Most of the respondents tended to attribute positive characteristics to the devices with regard to safety, reliability, effectiveness, ease of use and practicality. Although approximately 75% of the respondents indicated that an inhaler device is the best solution for respiratory diseases and 45% believed that inhaler devices could also be useful for other diseases, one-third of the respondents believed inhaler devices to be useful primarily for treating milder diseases and administering mild treatments. Moreover, approximately one-third of the respondents were unsure of how the device functions; this uncertainty may affect their adherence to treatment. This issue remains unsatisfactory; only one out of four individuals used the therapy regularly. These results are in line with data collected in clinical studies [13,17,29]. Approximately nine patients out of 10 stated that their physician provided an adequate explanation regarding the inhalation technique. However, data have also emerged from several studies that evaluated the occurrence of errors during inhaler device usage; a high proportion of patients do not have the competence to use their device effectively because they have forgotten what they were taught and no longer apply the correct technique that they were trained to use [30]. For this reason, international guidelines for asthma and COPD management state that inhalation technique should be assessed regularly and corrected if it is inadequate [11,12].

An increased rate of inhalation mistakes could be caused by device switches that are not supported by proper patient education. Although they prefer to maintain the same device when a change in dose or drug is necessary, device users must be informed about how a new device works, and they prefer to be informed by their physician. The need for a real partnership is underlined by most patients as a fundamental step during the switching of any device [31,32].

Compared with people who have never used an inhaler device, sufferers from asthma or COPD are more confident on inhaler device use, show less fear and show a greater sense of security, reliability and trust in this treatment option. People older than 64 years showed less attention to device properties than younger people.

## Conclusions

In conclusion, the awareness of asthma and COPD is unsatisfactory; despite their prevalence, these pathologies remain relatively unknown, and their management in sufferers appears to be far from ideal. One finding that emerges from this study is that inhaled drugs are recognised as the best solution for asthma and COPD because they offer positive characteristics of safety and usability. The public opinion that by these devices can only be used treat mild diseases with weak therapies should be addressed. The possibility that switching between devices could be associated with mistakes in drug consumption and a decrease in treatment adherence must be addressed by a proper educational plan.

## Competing interests

The authors declare that they have no competing interests.

## Authors’ contributions

FB, IB, FB and GWC designed the study, contributed to the analysis, to the interpretation of the findings, and to the writing of the manuscript. MS recruited the volunteers and attended to methodology and data analysis. All authors read and approved the final manuscript.
